# R&D investments and employment decisions as a function of enterprise size and regional population density before and during COVID-19

**DOI:** 10.3389/frma.2023.1107309

**Published:** 2023-02-23

**Authors:** Jarle Aarstad, Stig-Erik Jakobsen, Olav Andreas Kvitastein

**Affiliations:** The Mohn Centre for Innovation and Regional Development, Western Norway University of Applied Sciences, Bergen, Norway

**Keywords:** R&D investments, employment, enterprise size, regions, COVID-19

## Abstract

Norwegian data show that from 2018 to 2020, enterprises in densely populated regions increased R&D investments relative to those in sparsely populated regions, but not from 2016 to 2018. Therefore, COVID-19 likely induced the shift. The findings imply that densely populated regions have become more R&D-intensive, while sparsely populated regions have become less R&D-intensive during the pandemic. Small enterprises increased both R&D investments and employment from 2018 to 2020 relative to large enterprises and the analyses control for regression toward the mean effects. The findings were similar to those observed in the period from 2016 to 2018, which rules out COVID-19 as an explanation. Instead, the waves of data indicate a long-term trend where small enterprises increased R&D investments and employment.

## 1. Introduction

This article's major focus is to study how COVID-19 has affected Norwegian enterprises' decisions concerning R&D investments and employment. To do so, we measure enterprises' R&D investments and employment during the 1st year of COVID-19—in 2020—relative to 2018 by using data from different waves of Statistics Norway's Norwegian Community Innovation Survey. In addition, we study whether enterprise size in employees before COVID-19 and location in sparsely vs. densely populated regions have affected R&D investments and employment by comparing 2018 data and 2020 data during the pandemic. We also conduct similar pre-pandemic analyses to validate our results by comparing data between 2016 and 2018.

### 1.1. R&D investments

Research has examined how R&D investments may have reduced enterprises' negative impact of COVID-19 (Biswas, 2021; Behbahaninia and Golbidi, 2022) and has further debated how R&D investing enterprises have learned from the pandemic (Di Minin et al., 2021; Mortara et al., 2022). Moreover, research has suggested that enterprises have reduced R&D investments in the wake of COVID-19 as they did during the financial crisis (Roper and Turner, 2020).

Along these lines of research, it is not unlikely that the overall R&D investments may have been reduced due to the COVID-19 pandemic. However, it is also a matter of fact that while some enterprises were hit hard by the pandemic, others were less affected and even had a proactive response to it (Aarstad et al., 2022b). Therefore, we apply perceptual and revenue indicators in this study to assess whether negative or positive COVID-19 affectedness is linked to changes in R&D investments. An argument that counters a possible association between the concepts is that R&D investments, in their nature, have a long-term focus and are, therefore, unlikely to fluctuate much in the short run (Brown et al., 2012). However having said that, we do not rule out that R&D investments were subject to change during the COVID-19 pandemic since numerous enterprises experienced liquidity problems (Chebbi et al., 2021).

In addition to studying perceptual and revenue indicators of COVID-19 affectedness as potential carriers of changes in R&D investments, we also assess whether pre-COVID enterprise size in employees and location in densely vs. sparsely populated regions have played a role. Research has discussed how small- and medium-sized enterprises have coped with COVID-19 (Dai et al., 2021; Haneberg, 2021; Al-Maliki et al., 2022), but they are silent about whether size has affected R&D investments in the wake of the pandemic. We believe that owners and managers of large enterprises may have used COVID-19 as an excuse to reduce R&D investments in favor of stockholders' short-term gains, independent of the extent to which one has been affected or not by the pandemic. Therefore, we expect a negative association between enterprise size and R&D investments in the wake of COVID-19.

Concerning regional issues, we believe that COVID-19 may have reduced R&D investments in enterprises located in sparsely populated regions and increased R&D investments in enterprises located in densely populated regions. The reason is that COVID-19 may have induced a risk-averse attitude favoring R&D investments in densely populated regions with abundant relevant infrastructure, e.g., higher education institutions and other public and private enterprises involved in R&D (cf. Rypestøl and Aarstad, 2018; Aarstad and Jakobsen, 2019; Aarstad et al., 2022a). In other words, we assume that COVID-19 has propelled a Mathew effect of accumulative advantage (Merton, 1968) as densely populated regions with strong R&D milieus have further strengthened their position.

### 1.2. Employment

We study the association between our second effect variable, employment in 2020—the 1st year of COVID-19—relative to 2018, and the same independent variables we addressed earlier. It is intuitive—and in line with other research (Islam, 2021)—to assume that enterprises strongly affected by COVID-19 have reduced their employment, but having said that we are less sure whether employment reduction overall has been marked more or less among small vs. large enterprises and whether there are regional differences. However, similar to what we have argued earlier, we believe that owners and managers of large enterprises may have used COVID-19 as an excuse to reduce employment in favor of stockholders' short-term gains, independent of whether one has been affected or not by the pandemic. Therefore, we expect a negative association between enterprise size and employment in the wake of COVID-19. Concerning regional issues, we are less sure about any specific association and take an explorative approach to measure the concept's potential effect on enterprises' employment decisions in the wake of COVID-19.

### 1.3. Pre-pandemic comparative analyses

Finally, we compare two pre-COVID periods, 2016 and 2018 (except for the mentioned perceptual measures concerning COVID-19 affectedness). Our motive is to assess whether the 2016–2018 and 2018–2020 results converge or diverge. If they diverge, those between 2018 and 2020 are likely to be attributed to COVID-19, but not if they converge.

## 2. Materials and methods

### 2.1. Research context

To study our research questions, we used different waves of the Community Innovation Survey (CIS), carried out biannually by Statistics Norway on enterprises as the legal unit. It covers most industries and all regions of the country, and participation is mandatory, which minimizes the problem of non-respondent bias. All enterprises with 50 or more employees participate, and for those that are smaller, strata of enterprises of different sizes are selected for participation, which varies for different waves of the survey. The CIS includes enterprises with at least five employees only, and the respondent is normally the CEO, the deputy CEO, or the person responsible for innovation activities.

### 2.2. Dependent and independent variables

To measure the change in R&D investments between 2018 and 2020 as a dependent, we subtracted the logarithmic value of enterprises' 2018 total R&D investments from the logarithmic value of the same enterprises' 2020 total R&D investments (using the natural logarithm). That is, as the study includes numerous variables of changes in values, it only includes enterprises participating in both waves of the CIS. Moreover, it only includes enterprises reporting R&D investments in both waves. All monetary concepts are measured in 2020 values by using Statistics Norway's consumer price inflator. Change in employees between 2018 and 2020 as another dependent variable and change in revenues between 2018 and 2020 as an independent variable, we modeled similarly as a change in R&D investments.

The 2020 CIS included five items reflecting the extent to which enterprises were affected negatively or positively by COVID-19, and we list them in Table 1 (all translations from Norwegian are ours). Concerning each item, the respondent could indicate to a great extent (coded 4), to some extent (coded 3), to a small extent (coded 2), and to no extent (coded 1). We carried out principal component factor analysis with orthogonal varimax rotation, which identified the two factors we reported in Table 1. The eigenvalue for two factors is 1.56, explaining 69.2% of the variance (and the eigenvalue for three factors is 0.674), showing satisfactory convergent and divergent validity. Cronbach's alpha scores show satisfactory reliability for the indicators identifying each factor. To measure them, we took the respective items' average score, which we reported in bold.

**Table 1:** Principal component factor analysis with an orthogonal varimax rotation.

**Factors 1 and 2, respectively**	**Positively affected by COVID**	**Negatively affected by COVID**
Did the enterprise strengthen its position compared to the competitors due to the situation concerning COVID-19?	**0.833**	−0.040
Has the enterprise had commercial gains due to the situation concerning COVID-19?	**0.794**	−0.275
Has the enterprise become more efficient due to the situation concerning COVID-19?	**0.687**	0.273
Has the enterprise experienced economic consequences due to the situation concerning COVID-19 that will affect the enterprise negatively in the long term?	−0.099	**0.872**
Did the enterprise lose competitiveness due to the situation concerning COVID-19?	0.000	**0.862**
*Cronbach's alpha for items in bold*	*0.666*	*0.714*

*N* = 716.

We used the log-transformed number of employees in 2018 to model enterprise size as an independent variable. As another independent variable, we measured regional population density by dividing the number of inhabitants in 2020 in each of Norway's 85 economic regions by geographic size in square kilometers and then log-transformed the variable. Statistics Norway provided data on the 85 regions' geographic size and the number of inhabitants, and other studies measure regional population density similarly as we do (e.g., Aarstad et al., 2016).

### 2.3. Control variables

As control variables, we included R&D investments per employee in 2018 and productivity in 2018. The first variable was measured by dividing R&D investments by the number of employees, and the second variable was measured by dividing turnover by the number of employees. Both variables were log-transformed. Our motive for including R&D investments per employee is to account for a potential regression toward the mean effect, i.e., relatively high R&D investments one year may, ceteris paribus, be lower the next year and vice versa. Our motive for including productivity is that productive enterprises, ceteris paribus, will increase the number of employees at a later stage and vice versa. For consistency, we included both control variables when modeling change in R&D investments and change in employment as dependent variables. Finally, we control for the change in revenues, employment, and R&D investments between 2016 and 2018. We model the variables as described earlier (concerning changes between 2018 and 2020). Our motive for including the control variables is to further account for potential regression toward the mean effects and unobserved heterogeneity that the other control variables do not account for. In addition, we replicated all models by including dummies that control for the enterprises' largest market (regionally, nationally, in Europe outside of Norway, or outside of Europe) but without altering any statistical conclusion that we will report. Shortly, we address how we control industry effects.

### 2.4. Pre-pandemic comparative analyses

Concerning the variables to do comparative analyses between two pre-COVID periods, 2016 and 2018, we follow a similar procedure to measure them as described earlier, except that the timeline is 2 years back in time. Understandably, these analyses do not include perceptual variables of COVID-19 affectedness.

## 3. Results

### 3.1. Descriptive statistics and correlations

Table 2 reports descriptive statistics, and Tables 3, 4 report correlations. It is farfetched to comment on all the numbers, but Table 2 shows a more marked reduction in R&D investments from 2016 to 2018 than from 2018 to 2020, suggesting that COVID-19 has not reduced R&D investments. Enterprise employment increased more from 2016 to 2018 than from 2018 to 2020, which indicates that COVID-19 may have induced a relative reduction. Finally, we note that revenues increased from 2016 to 2018 but decreased from 2018 to 2020, and a likely interpretation is that COVID-19 induced a negative shift.

**Table 2:** Descriptive statistics, 2018–2020 and 2016–2018.

	**2018–2020**			**2016–2018**		
	**Mean**	**SD**	***N***	**Mean**	**SD**	***N***
Ch. in R&D 18-20/16-18 (log)	−0.072	1.47	757	−0.240	1.27	650
Ch. in empl. 18-20/16-18 (log)	0.015	0.279	757	0.055	0.244	650
Ch. in rev. 18-20/16-18 (log)	−0.074	0.831	757	0.240	0.680	650
Pos. affected by COVID 20	2.2	0.643	753			
Neg. affected by COVID 20	2.04	0.711	753			
Size in empl. 18/16 (log)	4.14	1.22	757	4.20	1.25	650
Reg. pop. dens. 20/18 (log)	4.88	1.96	757	4.87	1.89	650
R&D per empl. 18/16 (log)	4.32	1.77	757	4.55	1.62	650
Productivity 18/16 (log)	7.89	0.917	757	7.81	1.00	650
Ch. in rev. 16-18/14-16 (log)	0.294	0.784	757	0.085	0.691	650
Ch. in empl. 16-18/14-16 (log)	0.087	0.251	757	0.039	0.270	650
Ch. in R&D 16-18/14-16 (log)	−0.191	1.31	757	0.134	1.18	650

**Table 3:** Pairwise correlations, 2018–2020.

**1. Ch. in R&D 18-20 (log)**	**1**	**2**	**3**	**4**	**5**	**6**	**7**	**8**	**9**	**10**	**11**
2. Ch. in empl. 18-20 (log)	0.177^***^										
3. Ch. in rev. 18-20 (log)	0.014	0.207^***^									
4. Pos. aff. by COVID 20	−0.001	0.033	0.086^*^								
5. Neg. aff. by COVID 20	−0.040	−0.146^***^	−0.113^**^	−0.093^*^							
6. Size in empl. 18 (log)	0.035	−0.167^***^	0.006	0.090^*^	−0.118^**^						
7. Reg. pop. dens. 20 (log)	0.077^*^	−0.0175	−0.068^†^	0.004	0.027	0.000					
8. R&D per empl. 18 (log)	−0.354^***^	0.022	−0.069^†^	−0.045	0.077^*^	−0.455^***^	0.226^***^				
9. Productivity 18 (log)	0.024	0.063^†^	−0.268^***^	0.020	−0.134^***^	0.356^***^	−0.066^†^	−0.226^***^			
10. Ch. in rev. 16-18 (log)	−0.007	0.076^*^	−0.276^***^	0.018	0.034	−0.143^***^	0.118^**^	0.183^***^	0.064^†^		
11. Ch. in empl. 16-18 (log)	0.053	0.147^***^	0.128^***^	0.051	−0.003	−0.066^†^	−0.019	0.060^†^	−0.123^***^	0.318^***^	
Ch. in R&D 16-18 (log)	−0.473^***^	−0.034	−0.038	0.006	0.022	0.026	−0.003	0.438^***^	0.047	0.031	0.051

We report conservative two-tailed tests of significance.

† *p* < 0.10;

* *p* < 0.05;

** *p* < 0.01;

*** *p* < 0.001.

**Table 4:** Pairwise correlations, 2016–2018.

**1. Ch. in R&D 16–18 (log)**	**1**	**2**	**3**	**4**	**5**	**6**	**7**	**8**	**9**
2. Ch. in empl. 16–18 (log)	0.116^**^								
3. Ch. in rev. 18–20 (log)	0.004	0.262^***^							
4. Size in empl. 16 (log)	−0.025	−0.165^***^	−0.161^***^						
5. Reg. pop. dens. 18 (log)	−0.034	−0.040	0.075^†^	−0.005					
6. R&D per empl. 16 (log)	−0.276^***^	0.123^**^	0.169^***^	−0.477^***^	0.231^***^				
7. Productivity 16 (log)	0.010	−0.012	−0.443^***^	0.368^***^	−0.113^**^	−0.296^***^			
8. Ch. in rev. 14–16 (log)	0.036	0.175^***^	0.046	−0.094^*^	0.046	0.165^***^	0.059		
9. Ch. in empl. 14–16 (log)	0.117^**^	0.191^***^	0.159^***^	−0.025	−0.027	0.061	−0.141^***^	0.411^***^	
Ch. in R&D 14–16 (log)	−0.326^***^	0.077^*^	0.081^*^	0.043	−0.022	0.271^***^	−0.029	0.052	0.129^**^

We report conservative two-tailed tests of significance.

† *p* < 0.10;

* *p* < 0.05;

** *p* < 0.01;

*** *p* < 0.001.

Concerning the correlations reported in Tables 3, 4, it is worth noting that the association is practically zero between enterprise size and regional population density. It means that enterprises of different sizes in our data are equally distributed across regions of different population densities. On the contrary, enterprises investing much in R&D are predominately located in densely populated regions, as observed by the positive correlation between the concepts. Finally, it is interesting to note that productive enterprises invest relatively little in R&D, and vice versa, as observed by the negative correlation, which is in line with the exploration vs. exploitation dichotomy popularized by March (1991).

### 3.2. Regressions

Table 5 reports multi-level mixed-effects random intercept regression models where enterprises operating in different industries, according to their digit-two classification that follows the NACE-system, are nested in economic regions (for further details, please see, Raudenbush and Bryk, 2002; Snijders, 2011). That is, we account for potential industry effects and even for operating in a particular industry in a particular region.

**Table 5:** Multi-level mixed-effects random intercept linear regressions, 2018–2020.

	**Model 1**	**Model 2**	**Model 3**	**Model 4**	**Model 5**
**Dependent variable**	**Ch. in R&D 2018–2020 (log)**	**Ch. in empl. 2018–2020 (log)**	**Ch. in rev. 2018–2020 (log)**	**Pos. affected by COVID**	**Neg. affected by COVID**
**Fixed effects**					
Intercept	0.499	−0.156	1.84^***^	2.02^***^	2.91^***^
	(0.576)	(0.119)	(0.301)	(0.260)	(0.285)
**Independent variables**					
Ch. in rev. 2018-2020 (log)	−0.034	0.086^***^			
	(0.061)	(0.013)			
Pos. affected by COVID	−0.021	0.004			
	(0.072)	(0.015)			
Neg. affected by COVID	−0.051	−0.046^**^			
	(0.066)	(0.014)			
Size in empl. 2018 (log)	−0.098^*^	−0.059^***^	0.027	0.049^*^	−0.046^†^
	(0.046)	(0.010)	(0.028)	(0.024)	(0.026)
Reg. pop. density 2020 (log)	0.103^***^	0.002	−0.014	0.003	0.015
	(0.024)	(0.005)	(0.015)	(0.017)	(0.016)
**Control variables**					
R&D per empl. 2018 (log)	−0.218^***^	−0.006	−0.026	−0.005	−0.001
	(0.036)	(0.007)	(0.021)	(0.018)	(0.020)
Productivity 2018 (log)	0.038	0.064^***^	−0.230^***^	−0.003	−0.094^**^
	(0.057)	(0.012)	(0.033)	(0.028)	(0.031)
Ch. in rev. 2016-2018 (log)	−0.021	0.026^†^	−0.321^***^	0.015	0.038
	(0.067)	(0.014)	(0.038)	(0.032)	(0.036)
Ch. in empl. 2016-2018 (log)	0.544^**^	0.110^**^	0.657^***^	0.128	−0.082
	(0.198)	(0.041)	(0.117)	(0.099)	(0.108)
Ch. in R&D 2016-2018 (log)	−0.402^***^	−0.003	−0.002	0.004	0.018
	(0.041)	(0.008)	(0.025)	(0.021)	(0.023)
**Random effects**					
Residual	1.56	0.067	0.563	0.396	0.464
	(0.081)	(0.003)	(0.029)	(0.023)	(0.029)
Nested industry effect	1.25e−7	1.59e−15	2.07e−14	0.004	0.025
	(6.27e−5)	(2.08e−11)	(2.93e−14)	(0.010)	(0.020)
Region effect	2.59e−9	2.21e−11	5.19e−9	0.009	0.002
	(1.40e−8)	(1.33e−10)	(2.97e−8)	(0.010)	(0.007)
Wald χ^2^	286.9^***^	127.6^***^	170.0^***^	9.06	22.5^**^
Log likelihood	−1234.2	−49.3	−856.8	−729.6	−799.1
Likelihood ratio (LR) χ^2^	0.00	0.00	0.00	1.83	3.20
Maximum/average VIF	1.91/1.31	1.91/1.37	1.90/1.36	1.91/1.36	1.91/1.36
Number of enterprises	752	752	757	753	753
Number of regions	77	77	77	77	77
Industr. nested in regions	409	409	411	410	410

We report standard errors in parentheses, and for fixed effects, we report conservative two-tailed tests of significance.

† *p* < 0.10;

* *p* < 0.05;

** *p* < 0.01;

*** *p* < 0.001.

The lower part of Table 5 shows that the different models include a little more than 750 enterprises investing in R&D and are included in 2016, 2018, and 2020 waves of the CIS. They are located in 77 of Norway's 85 economic regions.

Changes in R&D investments from 2018 to 2020 are the dependent variable in Model 1, and we observe a negative effect of size on employees in 2018. It implies that large enterprises have downscaled their R&D investments to a relative extent more than small enterprises (note that the study includes different control variables that account for potential regression toward the mean effects, which we have addressed earlier). In addition, we observe that localization in densely populated regions has induced a positive shift in R&D investments compared to localization in sparsely populated regions.

Model 2 shows that revenue changes between 2018 and 2020 have affected employment, which is expected. In other words, decreasing revenues have reduced employment and *vice versa*. Moreover, Model 2 shows that enterprises perceived to have been negatively affected by COVID-19 have reduced their employment, but those perceived to have been positively affected have not increased theirs. Finally, Model 2 reveals that large enterprises have reduced their work stock to a relative extent more than small enterprises, while localization in densely vs. sparsely populated regions has not affected changes in employment.

Additional analyses show that large enterprises perceive to have been less affected negatively by COVID-19 than small enterprises (Model 5). Moreover, the results indicate that large enterprises perceive to have been more positively affected than small enterprises (Model 4), but enterprise size has had no significant effect on how revenues changed from before and during COVID-19 (Model 3).

### 3.3. Pre-pandemic regressions

Table 6 replicates the aforementioned table's two first models except that it includes 2 years earlier data waves and, intuitively, excludes the COVID-19 affectedness and responsiveness variables. Model 1 shows, in line with the aforementioned analyses, that large enterprises have downscaled their R&D investments from 2016 to 2018 to a relative extent more than small enterprises, but localization in densely vs. sparsely population regions has had no effect.

**Table 6:** Multi-level mixed-effects random intercept linear regressions, 2016–2018.

	**Model 1**	**Model 2**
**Dependent variable**	**Ch. in R&D 2016–2018 (log)**	**Ch. in empl. 2016–2018 (log)**
**Fixed effects**		
Intercept	1.26^*^	−0.211^*^
	(0.512)	(0.100)
**Independent variables**		
Ch. in rev. 2016-2018 (log)	0.057	0.106^***^
	(0.073)	(0.015)
Size in empl. 2016 (log)	−0.154^***^	−0.033^***^
	(0.044)	(0.009)
Reg. pop. density 2018 (log)	0.020	−0.006
	(0.035)	(0.005)
**Control variables**		
R&D per empl. 2016 (log)	−0.245^***^	0.005
	(0.035)	(0.007)
Productivity 2016 (log)	0.017	0.048^***^
	(0.055)	(0.011)
Ch. in rev. 2014-2016 (log)	0.040	0.027^†^
	(0.073)	(0.015)
Ch. in empl. 2014-2016 (log)	0.656^***^	0.116^**^
	(0.188)	(0.037)
Ch. in R&D 2014-2016 (log)	−0.270^***^	0.007
	(0.041)	(0.008)
**Random effects**		
Residual	1.18	0.051
	(0.090)	(0.003)
Nested industry effect	0.094	1.28e−12
	(0.076)	(2.98e−12)
Region effect	0.039	1.81e−16
	(0.034)	(2.33e−12)
Wald χ^2^	152.7^***^	113.5^***^
Log likelihood	−1005.0	47.5
Likelihood ratio (LR) χ^2^	6.34^*^	0.00
Maximum/average VIF	1.63/1.34	1.63/1.34
Number of enterprises	650	650
Number of regions	75	75
Industr. nested in regions	375	375

We report standard errors in parentheses, and for fixed effects, we report conservative two-tailed tests of significance.

† *p* < 0.10;

* *p* < 0.05;

** *p* < 0.01;

*** *p* < 0.001.

Model 2 shows, also in line with the aforementioned analyses, that revenue changes between 2016 and 2018 have affected employment between the two same years, which is expected. In other words, decreasing revenues have reduced employment, and vice versa. Moreover, in line with previous analyses, Model 2 shows that large enterprises have downscaled their employment from 2016 to 2018 to a relative extent more than small enterprises. In the following section, we summarize and discuss the overall findings that we have reported.

## 4. Discussion and conclusion

Our primary focus in this article was to study how COVID-19 may have affected enterprises' decisions concerning R&D investments and employment. Second, we aimed to assess whether pre-COVID enterprise size in employment and location in densely vs. sparsely populated regions have played a role.

Using different waves of the Community Innovation Survey by Statistics Norway, we found that small enterprises increased both R&D investments and employment from 2018 to 2020 relative to large enterprises. The findings were similar from 2016 to 2018, likely ruling out COVID-19 as an explanation. Instead, the waves of data indicate a long-term trend where small enterprises have increased R&D investments and employment. From 2018 to 2020, enterprises in densely populated regions increased R&D investments relative to those in sparsely populated regions but not from 2016 to 2018. Therefore, COVID-19 likely induced the shift. The findings imply that densely populated regions with predominately small enterprises have become more R&D-intensive, while sparsely populated regions with predominately large enterprises have become less R&D-intensive.

Neither perceptual measures of COVID-19 affectedness nor revenue changes, e.g., because of COVID-19, affected changes in R&D investments between 2018 and 2020. Our findings contradict recent research suggesting that enterprises have reduced R&D investments in the wake of COVID-19 as they did during the financial crisis (Roper and Turner, 2020). Thus, our findings, or non-findings, may indicate that R&D investments have a long-term focus and are relatively insensitive to fluctuate due to short-term impulses affecting the enterprise (cf. Brown et al., 2012). A complementary explanation can be that governmental support has prevented R&D reductions, which future research should aim to investigate.

Concerning employment, on the contrary, we observe that decreasing revenues have had a negative effect, but the association is not particularly tailored to COVID-19, which is understandable. Therefore, enterprises losing money, independent of reason, need to reduce the work stock. We observe, however, that enterprises perceiving that they were negatively affected by COVID-19 reduced their work stock beyond the de facto effect of revenue losses.

Our study contributes to the growing literature addressing how enterprises navigate during crises in general and during the COVID-19 pandemic in particular. Of special interest for the research field and industry stakeholders are our observations that there now appears to be a shift in R&D investments and employment from relatively large to relatively small enterprises, but we cannot attribute it to COVID-19 since the trend was also apparent during periods before the exogenous shock. We argued that owners and managers of large enterprises may have used COVID-19 as an excuse to reduce R&D investments and employment in favor of stockholders' short-term gains, independent of the extent to which one has been affected or not by the pandemic. However, observing that these trends were also present before the pandemic counters our argument. A potential explanation is a path-dependent process, which “stresses the importance of past events for future action or, in a more focused way, of foregoing decisions for current and future decision-making” (Sydow et al., 2009, p. 690). In other words, an issue initiating a reduction in R&D investments and employment in a large enterprise at one point in time may have induced future reductions due to a path-dependent process, while the opposite is the case in smaller enterprises. Similarly, we know that enterprises often mimic each other (Di Maggio and Powell, 1983), implying that large (small) enterprises have adopted similar strategies as other large (small) enterprises by copying each other. Nonetheless, future research should investigate further potential explanations of why the trends occur. Moreover, assessing whether the trends will persist in the future is another topic for future research.

Assuming that the aforementioned trends persist, an implication is a shift in the landscape of enterprises of different sizes and R&D intensity. Small enterprises invest more in R&D per employee than large enterprises (Tables 3, 4), and observing that the trend is reinforcing will imply that they will play an important role as a vehicle for new and improved products, services, and production processes in the future. As small enterprises increase employment, becoming relatively larger may further propel the process.

Finding that R&D investments have shown a shift from enterprises located sparsely to densely populated regions, we attribute to COVID-19 as we did not observe a similar trend before the pandemic. A likely explanation is that COVID-19 may have induced a risk-adverse attitude inducing enterprises to channel such funding into densely populated regions with abundant relevant infrastructure, e.g., higher education institutions and other public and private enterprises involved in R&D (cf. Rypestøl and Aarstad, 2018; Aarstad and Jakobsen, 2019; Aarstad et al., 2022a). However, we are unsure whether ours is a valid explanation, and future research should aim to further explain the shift in R&D investments. For instance, we are unsure about potential long-term effects beyond 2020, and despite including relevant controls and having time asymmetry in the data, one should always be careful to make causal inferences. Moreover, future research should conduct follow-up studies to see whether the trend persists beyond COVID-19 and assess whether R&D investments, for instance, are used for incremental or radical innovation activities.

Other topics for future research would be to investigate how enterprises without R&D investments were affected by COVID-19 and assess the effects of the pandemic across industries and national borders. Finally, one should aim to assess how government support during COVID-19 may have affected the associations we have studied in this article as these data, unfortunately, are not available in the Community Innovation Study.

## Data availability statement

The data analyzed in this study is subject to the following licenses/restrictions: The data is restricted by Statistics Norway. Requests to access these datasets should be directed to jarle.aarstad@hvl.no.

## Author contributions

JA designed the study, analyzed the data, and wrote the manuscript. S-EJ assisted in designing the study and in editing the manuscript. OK assisted in analyzing the data and in editing the manuscript. All authors contributed to the article and approved the submitted version.
